# A systematically structured review of biomarkers of dying in cancer patients in the last months of life; An exploration of the biology of dying

**DOI:** 10.1371/journal.pone.0175123

**Published:** 2017-04-06

**Authors:** Victoria Louise Reid, Rachael McDonald, Amara Callistus Nwosu, Stephen R. Mason, Chris Probert, John E. Ellershaw, Séamus Coyle

**Affiliations:** 1 The Marie Curie Palliative Care Institute Liverpool, University of Liverpool, Liverpool, United Kingdom; 2 Renal Medicine, Aintree University Hospital NHS Foundation Trust, Liverpool, United Kingdom; 3 Department of Gastroenterology, University of Liverpool, Liverpool, United Kingdom; Taipei Medical University, TAIWAN

## Abstract

**Background:**

The Neuberger review made a number of recommendations to improve end of life care, including research into the biology of dying. An important aspect of the biology of dying is the identification of biomarkers as indices of disease processes. Biomarkers have the potential to inform the current, limited understanding of the dying process and assist clinicians in recognising dying, in particular how to distinguish dying from reversible acute deterioration.

**Objectives:**

To critically appraise the literature on biological factors that may be used as prognostic indicators in advanced cancer patients and to identify candidate biomarkers of the dying process that can be measured serially in cancer patients’ bodily fluids.

**Methods:**

A systematically structured review was conducted using three electronic databases. A hand search of six peer-reviewed journals and conference abstracts was also conducted. Studies reporting prognostic biomarkers in cancer patients with a median survival of ≤90 days and post-mortem studies were included. Final levels of evidence and recommendations were made using the Evidence Based Medicine modified GRADE system.

**Results:**

30 articles were included. Seven prognostic biological factors demonstrated *Grade A* evidence (lymphocyte count, white blood cell count, serum C-reactive protein, albumin, sodium, urea and alkaline phosphatase). An additional eleven prognostic factors were identified with *Grade B* evidence (platelet count, international normalised ratio, serum vitamin B12, prealbumin, bilirubin, cholesterol, aspartate aminotransferase, alanine transaminase, lactate dehydrogenase, pseudocholinesterase and urate). A number of biomarkers were specifically identified in the last two weeks of life but limitations exist. No post-mortem studies met the inclusion criteria.

**Conclusion:**

The biology of dying is an important area for future research, with the evidence focused on signs, symptoms and prognostic factors. This review identifies a number of common themes shared amongst advanced cancer patients and highlights candidate biomarkers which may be indicative of a common biological process to dying.

## Background

In a comprehensive evaluation of the challenges and actions required to provide the best care for dying patients, the Neuberger review identified the need for further research into the biology of dying as a priority [1]. The biology of dying is an umbrella topic that encompasses the physiological and biological changes attributed to the dying process, in addition to the aetiology of signs and symptoms commonly seen in the last days, weeks and months of life.

There is significant uncertainty in consistently and accurately identifying the dying phase (last days of life) and no definitive diagnostic criteria exist [1,2]. Little is known about the process of dying [1] and in the final days of life, new symptoms or exacerbation or recurrence of previously well-controlled symptoms can occur [3,4]. A prospective cohort study of 343 doctors found only 20% of prognostic estimates in hospice patients were accurate and that, overall doctors overestimated survival by a factor of five [5].

It has been extensively documented that cancer patients experience a sharp functional decline in the last months of life [6]. The prevalence of common terminal symptoms amongst patients with advanced cancer is suggestive of a common terminal trajectory; historically termed the “terminal cancer syndrome” [7]. Although the concept of the “terminal cancer syndrome” has been largely superseded, there is significant evidence for the prognostic importance of dyspnoea (Grade B), cancer anorexia-cachexia syndrome (CACS, Grade B), delirium (Grade B) and low performance status (Grade A) in advanced cancer [8]. A systematic review by Kehl *et al*. demonstrated that dyspnoea (56.7%), pain (52.4%), respiratory tract secretions (51.4%) and confusion (50.1%) were the most prevalent signs and symptoms that occur in the last two weeks of life [9]. Further, Hui *et al*. identified 13 signs highly predictive of death within three days [10,11].

An important aspect of the biology of dying is the identification of biomarkers as indices of disease processes. Biomarkers have the potential to inform the current, limited understanding of the process of dying and assist clinicians in recognising dying, in particular how to distinguish dying from reversible acute deterioration. In 2005, Maltoni *et al*. published evidence-based recommendations for a number of prognostic biological factors in advanced cancer patients [8]. Subsequently, a number of prognostic models have been developed that incorporate prognostic biomarkers [12]. No systematic reviews have been conducted that summarise the evidence for prognostic biomarkers in advanced cancer patients.

In this systematically structured review, the authors summarise the evidence for prognostic biomarkers in advanced cancer patients in the last months of life and extrapolate which biological processes are affected.

### Objectives

This systematically structured review was conducted to collate and critically appraise the literature on prognostic biomarkers in advanced cancer. The following questions formed the basis of this review:

1. What biological factors are prognostic in advanced cancer patients in the last days, weeks or months of life?
2. Can serial measurement of identified biomarkers detect the last days to weeks of life in advanced cancer patients?

## Methods

Given that no randomised controlled trials have been conducted on this research topic, a systematically structured review was conducted to ensure a replicable and systematic synthesis of the evidence. The review was structured according to the PRISMA standards for conducting a systematic review [13].

### Literature search methods

On 5^th^ February 2016, three electronic databases were searched (Medline, Scopus and Cochrane Database of Systematic Reviews) using combinations of the key words, described in Table 1. Limits were set to humans, adults (aged over 18 years of age) and publication between 1^st^ January 2000 and 5^th^ February 2016. Only published articles were sought. Two reviewers (VLR and SC) independently searched all designated databases for abstracts and titles. Agreement on inclusion was made by consensus. Additional articles were identified through a hand search of contents pages of the most recent issues (December 2014 to February 2016) of six relevant peer-reviewed Palliative Medicine journals: Cancer, BMJ Supportive and Palliative Care, Palliative Medicine, Journal of Palliative Care, Journal of Palliative Medicine, and Journal of Pain and Symptom Management. Grey literature was searched through citation tracking and conference abstracts from the European Association of Palliative Care World Congress 2016, European Association of Palliative Care World Congress 2015, the Marie Curie Research Conference 2015, the Arts and Science of Hospice Care Annual Conference 2015, the 5^th^ International Conference on Advance Care Planning and End of Life Care 2015, and the International Congress of Palliative Care 2014. The last literature search was conducted on 16^th^ June 2016. Institutional review board approval was not sought or required for this literature review.

**Table 1:** Search strategy for medline.

Query Number	Query Content
#1	death OR dying OR terminal care OR terminal illness OR “terminally ill” OR hospice* OR palliative care
#2	biolog* OR physiolog* OR pathophysiolog* OR patholog* OR biomarker* OR biologic marker* OR biological marker* OR biological factor* OR “biology of dying” OR prognostic* OR prognosis OR predict OR mortality OR “terminal cancer syndrome” OR “common terminal pathway” OR “common terminal trajectory” OR “final common pathway” OR “terminal syndrome”
#3	cancer OR tumor* OR tumour* OR neoplas* OR oncolog* OR carcinoma
#4	#1 AND #2 AND #3
#5	Limit #4 to humans, all adults (19 years plus), publication year 2000—current

*Truncation

### Eligibility criteria

The primary objective was the identification of prognostic biomarkers in advanced cancer patients in the last days, weeks or months of life. Biomarkers were defined as objective, quantifiable characteristics of biological processes [14] quantifiable in bodily fluids and tissues. Given that most cancer patients seem to follow a common terminal trajectory, [6] only patients with cancer were included in this study.

Diagnosing imminent death is often difficult and imprecise [1] and terminal physiological changes are often seen many weeks before death. Further, the definition of “*advanced cancer*” is often lacking in the literature. In line with the study by Maltoni *et al*. this review included only study populations with a median survival of ≤90 days [8] that, for the purpose of this study, defines the term “*advanced cancer*.” This criterion enabled us to capture biological factors predictive of dying over days, weeks and months of life. Post-mortem studies of cancer patients specifically looking at biomarkers of the dying process were also included.

All types of peer-reviewed evidence were included and a 16-year timeframe was selected to ensure comprehensive yet current coverage of the literature, and build on the excellent review published in 2005 by Maltoni *et al* (last literature search conducted in 2003).

Articles were excluded if they described only signs, symptoms or physiological changes associated with imminent death. The following types of articles were also excluded: duplicates, non-English language, paediatric populations, editorials, commentaries, case reports, reviews and animal studies. The systematic review by Maltoni *et al*. was included as it provided an evidence-based summary of the literature up until 2005 [8]. Primary researchers were contacted by email, where necessary, to clarify information to ensure strict adherence to the inclusion criteria. Where survival data could not be confirmed, studies were excluded.

### Study selection

A review protocol was developed by VLR in advance of the literature search (S2 File). VLR extracted the data from the studies and discussed the results with SC. Disagreements between reviewers were resolved by consensus. Reviewers were not blinded for authors, institutions, or journals of publication. The Liverpool Reviews and Implementation Group (LR*i*G) at University of Liverpool reviewed and agreed on the employed methodology.

### Quality assessment

The primary authors (VLR and SC) used the UK National Institute for Health and Clinical Excellence (NICE) hierarchy of evidence to assign a quality rating to the potential articles for inclusion [15]. Given that a variety of research outputs were considered in this review, a critical appraisal tool described by Hawker *et al*. [16] was utilised to further evaluate the quality of studies. Briefly, a four-point scale from one (very poor) to four (good) was assigned to nine areas including: abstract and title, introduction and aims, methods and data, sampling, data analysis, ethics and bias, results, transferability/generalisability, and implications and usefulness with a total number between 9–36 assigned to each study [16]. The Maltoni *et al*. seven-point checklist of quality criteria for evaluation of studies on prognostic factors was also utilised, where one point is assigned to seven criteria including; prospective study design; well-defined cohort of patients assembled at a common point in the course of their disease; random patient selection, percentage of patients lost to follow-up ≤20%; ratio between the number of events (death) and the number of potential predictors ≥10; prognostic variables fully defined, accurately measured, and available for all or a high proportion of patients; and reliable measurement of outcome (date of death) [8]. A total score between 1–7 was assigned and high quality (or low probability of bias) was attributed to studies fulfilling at least five of the seven criteria [8]. VLR and SC assessed the quality of potential articles for inclusion independently and mean scores were assigned. Spearman’s rank correlation coefficient was used to measure rank correlations of scores between assessors. Data was analyzed using Statistical Software Package for the Social Sciences^®^ (SPSS^®^ version 22.0; IBM SPSS Inc., Chicago, IL). Final levels of evidence and recommendations were made using the Evidence Based Medicine modified Grading of Recommendations Assessments, Development and Evaluation (GRADE) system (Grade A = high quality evidence with consistent results, to Grade D = very low quality evidence such as expert opinion) [17].

## Results

### Study selection

**Fig 1:**
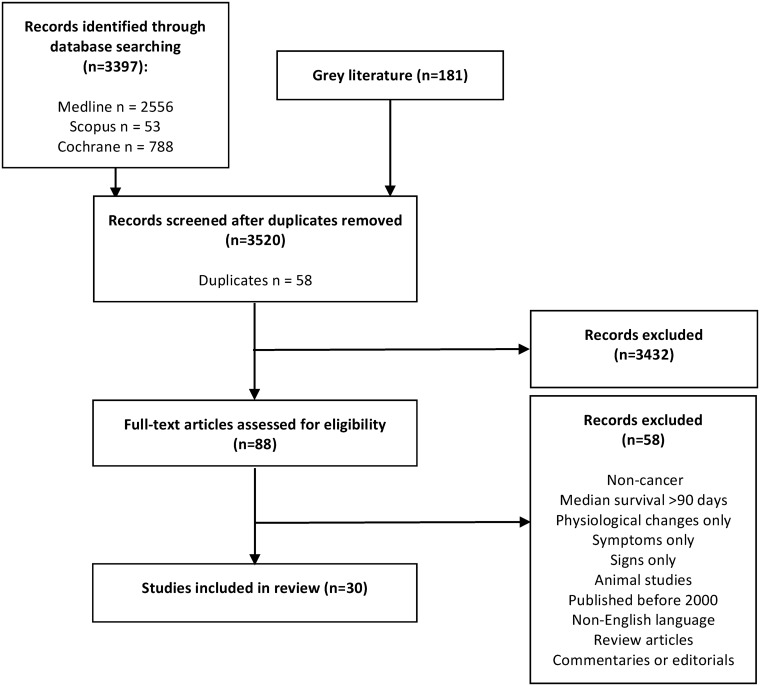
PRISMA flow diagram of this systematically structured review.

The results of the literature search are summarised in Fig 1. Based on firm application of the inclusion criteria, 30 articles were included in this systematically structured review (Table 2). A selected number of articles were excluded as patient populations had a median survival >90 days. An additional three articles were excluded due to lack of survival data.

**Table 2:** Summary of included studies.

Citation (author, year, country)	Design & Objectives	Setting and Sample	Diagnosis	Days until Death	Weaknesses	Appraisal	Main Study Findings
Geissbűhler, P *et al*. 2000, Switzerland [18]	Prospective cohort study (investigative) Assess the relationship between an elevated serum B12 and an earlier death. To assess if an elevated serum B12 predicted death independently of the PINI calculation and its derivatives: CRP, AGP, albumin, and prealbumin.	University hospital inpatients admitted to a geriatric facility requiring inpatient palliative care n = 161	Various malignancies Terminally ill Median age 76 years (range 46–96)	Median survival: 45 days (95% CI 32–56 days)	Relied on blood samples taken on admission (missing data: n = 4) Does not clearly distinguish between palliative and curative patients Requires invasive procedure	Level 2++ *Hawker et al*: 33/36 *Maltoni et al*: 6/7	• The length of survival decreased with the increase in serum B12 levels (p = 0.0015, Cox model). • In univariate analysis, PINI and its 4 components: CRP, AGP, albumin, prealbumin were predictors of length of survival. • In multivariate analyses, CRP was the most important prognostic factor, and B12 was independent of CRP in predicting survival. • There was a strong correlation between the presence of metastasis, hepatic dysfunction and an elevated serum B12 level (p<0.001). • The BCI, calculated by B12 x CRP, was a simplified and significant marker of survival (trend test, p<0.001): ○ *BCI <10000*: 50% 3 month mortality ○ *BCI >40000*: 90% 3 month mortality
Pasanisi, F *et al*. 2001, Italy [19]	Prospective cohort study (investigative) Evaluation of clinical, anthropometric, hematologic, and biochemical variables immediately before starting nutritional treatment and relationship to survival in terminal cancer patients with irreversible bowel obstruction receiving home parenteral nutrition.	Hospital patients in 2 centres in Italy n = 76	Terminally ill cancer patients with irreversible bowel obstruction receiving home parenteral nutrition Various malignancies Mean age: 56.8 years (SD 14.0)	Median survival: 74 days (range 6–301)	Ratio between the number of events (death) and the number of potential predictors <10 Method for identifying date of death not described Requires invasive procedure	Level 2+ *Hawker et al*: 29/36 *Maltoni et al*: 4/7	• Serum albumin (p = 0.001) emerged (with a positive sign) as predictive of survival. • *Statistically non significant*: ○ Lymphocyte count ○ Serum cholesterol
Glare, P *et al*. 2001, Australia [20]	Prospective cohort study (confirmative) External validation of the PaP Score in a population of hospitalised patients in Australia	University teaching hospital inpatients referred to a hospital-based palliative medicine consultation service in Australia n = 100	Terminally ill Advanced cancer, n = 91 Non-cancer, n = 9 Median age: 66.5 years (range 16–92)	Median survival: 30 days (95% CI: 24–40)	Includes non-cancer patients (n = 9) PaP Score requires invasive procedure	Level 2++ *Hawker et al*: 33/36 *Maltoni et al*: 6/7	• Pirovano *et al*. published details of the PaP Score, which classifies patients with very advanced cancer into homogeneous risk groups for survival based on various clinical and laboratory parameters (anorexia; dyspnoea; performance status; CPS; WBC; and lymphocyte count) [21] • The PaP Score subdivides the population into 3 groups with a different probability of survival at 30 days: ○ *Group A*: >70% ○ *Group B*: 30–70% ○ *Group C*: <30% [21] • This study externally validates the PaP Score in patients referred to the hospital palliative care team. Median survivals for the three groups were: ○ *Group A*: 60 days (95% CI 41–89) ○ *Group B*: 34 days (95% CI 25–40) ○ *Group C*: 8 days (95%CI 2–11) • The percentage survival at 30 days for the three groups were: ○ *Group A*: 66% ○ *Group B*: 54% ○ *Group C*: 5%
McMillan, DC *et al*. 2001, UK [22]	Cohort study (explorative) To assess the value of the incidental measurement of serum CRP and albumin concentrations as prognostic factors in a large cohort of hospital inpatients with solid tumors.	Hospital inpatients n = 404 Only bronchogenic cancer patients included, as the median survival >90 days in the remaining patient groups	Advanced bronchogenic cancer Median age: 66 years (range 39–95)	• Median survival: ○ Bronchogenic: 60 days	No protocol for blood sampling Does not distinguish between palliative and curative patients Clinicopathological stage at diagnosis and subsequent treatment were not included in analysis Requires invasive procedure High risk of bias	Level 2+ *Hawker et al*: 26/36 *Maltoni et al*: 3/7	On univariate analysis, log10 CRP and albumin concentrations were significant predictors of survival. On multivariate analysis, log10 CRP remained a significant independent predictor of survival. CRP concentrations were associated with a higher risk of non-cancer deaths.
Faris M. 2003, Oman [23]	Prospective cohort study (explorative) To analyse the pattern of various prognostic factors in terminally ill cancer patients in the only tertiary oncology centre in Oman.	Tertiary oncology centre in Oman n = 162	Terminally ill Solid organ tumours Mean age: 50 years (range 16–83)	Median survival: 10 days	Exclusion criteria not described Requires invasive procedure	Level 2+ *Hawker et al*: 30/36 *Maltoni et al*: 4/7	• *Univariate analysis demonstrated that a number of factors had a significant effect on survival including*: ○ Peripheral oedema ○ Low lymphocyte count ○ Low serum albumin level • *Multiple regression analysis demonstrated that the following as independent predictors of survival*: ○ Peripheral oedema (p = 0.007)
Ho SY *et al*. 2003, Taiwan [24]	Prospective cohort study (investigative) To evaluate relationships between various nutritional indices and the survival of terminally ill cancer patients.	University hospice in Taiwan n = 109	Terminally ill Advanced cancer Mean age: 65.3 years (range 32–95)	Median survival: 19 days	Limitations of study not discussed Serum albumin and lymphocyte count, previously identified prognostic markers, were not significant	Level 2++ Hawker *et al*: 32/36 Maltoni *et al*: 6/7	• *Univariate analysis demonstrated that a number of factors had a significant effect on survival including*: ○ Prealbumin ≤100 mg/L (p = 0.03) ○ AST > 45 U/L (p = 0.03) ○ ALP > 120 U/L (p = 0.02) ○ Serum creatinine >1.4mg/dL (p = 0.01) ○ BUN >20mg/dL (p<0.01) • *Multivariate analysis demonstrated that a number of factors were independent predictors of poor survival*: ○ Prealbumin ≤100 mg/L (p<0.01) ○ AST > 45 U/L (p = 0.01) ○ BUN > 20 mg/dL (p<0.01 • *Statistically non significant*: ○ Serum albumin ○ Transferrin ○ Haemoglobin ○ Total lymphocyte count ○ Serum ALT
Iwase S, *et al*. 2004, Japan [25]	Prospective cohort study (investigative) Quantitative serial analysis of six cytokines in the plasma: TNFα, IL-1β, IL-6, IFNγ, PTHrP and LIF and basic laboratory tests in hospice inpatients with late stages of cachexia.	Hospice inpatients in a single centre in Japan n = 28	• Various carcinomata ○ *Palliative*: n = 25 ○ *Curative*: n = 3 • Late stages of cachexia • Mean age: 65.2 years (range 38–90)	1–92 days Survival >90 days: n = 1	Small sample size Heterogeneity of sample No protocol for collection of blood samples Primary outcomes not specified Main findings were found incidentally Includes only patients with late stages of cachexia Ratio between the number of events (death) and the number of potential predictors <10 Requires invasive procedure Prognostic significance not assessed	Level 2+ *Hawker et al*: 31/36 *Maltoni et al*: 4/7	Only IL-6 (and TNFα, n = 1) was detected in all patients. Maximum plasma IL-6 4.6–870 pg/mL Concentration of IL-6 rose gradually during the early stages of cachexia. Sharp rise in the week prior to death (n = 10). Plasma levels of IL-6 ≥100 pg/mL detected in the week prior to death (n = 6).
Maltoni M *et al*. 2005, Italy [8]	Systematic review of non-randomised controlled trials Working group review of 38 prospective studies evaluating prognostic factors in advanced cancer. To offer evidence-based clinical recommendations concerning prognosis in advanced cancer patients.	38 studies	Advanced cancer Median survival ≤90 days Mean age: 65.2 years (range 38–90)	Median survival: ≤90 days	Heterogeneity of studies Meta analysis not possible Poor quality of existing published studies Some subjectivity in quality criteria	Level 2++ *Hawker et al*: N/A *Maltoni et al*: N/A	• *Level B evidence based recommendations for the prognostic correlation of a number of factors including*: ○ Leukocytosis ○ Lymphopaenia ○ Elevated serum CRP level ○ CACS • *Factors that proved significant in at least one multivariate analysis included*: ○ Low pseudocholinesterase ○ High serum B12 ○ High serum bilirubin • *Factors for which a correlation has been indicated but not confirmed or for which a statistical significance has been identified in patient populations with less advanced disease or for which contradictory data have emerged*: ○ Anaemia ○ Hypoalbuminaemia ○ Low prealbumin ○ Proteinuria ○ Serum calcium level ○ Serum sodium level ○ Serum LDH
Shin, HS *et al*. 2006, Korea [26]	Prospective cohort study (investigative) Serial measurements of a number of serological variables in terminally ill cancer patients admitted to a palliative care unit in a single inpatient centre.	Hospital inpatients admitted to the palliative care unit in a single centre in Korea n = 118	Various carcinomata Terminally ill Median age: 65.5 years (range 25–93)	Median survival: 14 days (95% CI 11–17)	Requires invasive procedure Single centre in Korea Ratio between the number of events (death) and the number of potential predictors <10	Level 2++ *Hawker et al*: 35/36 *Maltoni et al*: 5/7	• *Significantly shorter survival times were observed in the following groups in univariate analysis*: ○ Elevated serum urate ≥7.2mg/dL (p <0.001) ○ Increasing or decreasing leukocytes (p <0.001) ○ Hypercreatininaemia >1.5mg/dL (p = 0.006) ○ Low serum albumin <3.0g/dL (p = 0.026) ○ Hyperbilirubinaemia >1.0mg/dL (p = 0.001) ○ INR >1.12 (p < 0.001) ○ Hypocholesterolaemia <130 mg/dL (p = 0.016) ○ High serum LDH >378IU/L (p = 0.001) • *By multivariate analysis*, *the following variables significantly shortened survival*: ○ High serum uric acid concentration (HR 2.637, p = 0.001) ○ Prolonged INR (HR 1.686, p = 0.039) ○ Hypocholesterolaemia (HR 2.030, p = 0.011) ○ High serum LDH (HR 2.136, p = 0.001) • Patients were divided into 4 groups based on serum urate levels. Median survival for each group was: ○ *Group 1*, *serum urate <3*.*2mg/dL*: 18 days (955 CI 15–21) ○ *Group 2*, *serum urate ≥3*.*2 - <4*.*9mg/dL*: 23 days (95% CI 9–37) ○ *Group 3*, *serum urate ≥4*.*9 - <7*.*2mg/dL*: 12 days (95% CI 7–17) ○ *Group 4*, *serum urate ≥7*.*2mg/dL*: 4 days (95% CI 5–11) • In univariate analysis, survival time of the fourth highest group (serum urate ≥7.2mg/dL) was significantly shorter than that of other groups (HR 2.784, p <0.001). There was no significant difference amongst the first 3 groups. • The serum urate concentration was inversely associated with survival time (HR 2.778, p = 0.003). • Serum urate concentration was measured consecutively for 3 weeks before death (n = 39) and was significantly increased between the first and the second week before death (p = 0.006).
Lam PT *et al*. 2007, China [27]	Prospective cohort study (explorative) To identify potential prognostic factors affecting the survival in patients with advanced cancer who were treated in a palliative care unit at a regional hospital in Hong Kong.	Hospital inpatients and outpatients at a palliative care unit at a regional teaching hospital in Hong Kong n = 170	• Advanced cancer in the last 6 months of life: ○ Locally advanced (n = 28) ○ Metastatic (n = 142) • Various malignancies • Mean age: 69 years (SD 12)	Median survival: 77 days (range 31–160)	Confined to a single centre in Hong Kong Requires invasive procedure	Level 2++ *Hawker et al*: 30/36 *Maltoni et al*: 6/7	• By univariate analysis (Cox), a number of factors affected survival including: serum albumin concentration and WBC. • *Multivariate analysis (Cox) identified a number of factors were independent predictors of survival including*: ○ Serum albumin concentration (HR 0.95, 95% CI 0.92–0.98) • *Statistically non significant factors by univariate analysis*: ○ Serum calcium ○ Serum sodium ○ Haemoglobin
Kelly, L *et al*. 2007, UK [28]	Prospective cohort study (confirmative) Confirmatory study of the BCI	Hospital and hospice patients receiving palliative care in 7 UK centres n = 329	• Advanced cancer: ○ Locally advanced (n = 20) ○ Metastatic (n = 309) • Palliative • Median age 68.7 years (range 23–94)	Median survival: 42 days (95% CI 34.8–49.2)	Relevant blood tests not taken and excluded (n = 50) Patients censored due to missing data (n = 9) Non-consecutive series of patients No clinician estimate of survival Requires invasive procedure	Level 2++ *Hawker et al*: 35/36 *Maltoni et al*: 6/7	• The BCI, calculated by B12 x CRP is a simplified and significant marker of survival: ○ BCI < 10000: 50% 3 month mortality ○ BCI > 40000: 90% 3 month mortality [18] • In this study, the BCI was externally validated in hospital and hospice patients receiving palliative care. Patients were divided into 3 groups according to BCI score. The median survival for each group was: ○ *Group 1*, *BCI ≤10000*: 71 days (95% CI 45.9–96.2) ○ *Group 2*, *BCI 10001–40000*: 43 days (95% CI 28.8–57.2) ○ *Group 3*, *BCI >400000*: 29 days (95% CI 22.0–36.1) • Patients in Group 3 had a significantly worse survival than patients in Group 2 and patients in Group 1 (p < 0.01). • However, patients in Group 1 did not have a significantly better prognosis than those in Group 2 (p = 0.091). Thus, while a high BCI score had poor prognostic significance, an intermediate or low score was more difficult to interpret. • 90-day mortality estimates for each of the three risk groups were different from the figures previously reported: ○ *Group 1*, *BCI ≤10000*: 58.9% vs. 47.2% ○ *Group 2*, *BCI 10001–40000*: 64.0% vs. 72.5% ○ *Group 3*, *BCI >400000*: 78.9% vs. 90.6%
Suh, SY et al. 2007, South Korea [29]	Prospective cohort study (investigative) Serial measurements of serum LDH and other serological variables in terminal cancer patients admitted to a hospital palliative care unit.	Hospital palliative care unit in a single centre in South Korea n = 93	Various carcinomata Terminally ill Median age: 65 years (range 30–87)	Median survival: 19 days (95% CI 14–24)	Serum LDH concentrations may fluctuate with the passage of time or may reflect complications of cancer The relationship between serum LDH and survival time is not a simple negative correlation, and requires further research Preliminary study only due to sample size Single centre Requires invasive procedures	Level 2+ *Hawker et al*: 34/36 *Maltoni et al*: 4/7	• *Significantly shorter survival times were observed in the following groups in univariate analysis (Cox)*: ○ High serum LDH ≥313IU/L (p < 0.001) ○ Increased leukocytes >11000 mm^3^ (p = 0.020) ○ Increased neutrophil fraction >75% (p = 0.030) ○ Decreasing thrombocytes (p = 0.030) ○ Elevated serum CRP ≥9.5 mg/dL (p = 0.015) ○ Elevated serum urate ≥7.2 mg/dL (p <0.001) ○ Low serum albumin <3.0g/dL (p = 0.019) ○ Hyperbilirubinaemia >1.0mg/dL (p = 0.003) ○ INR >1.12 (p = 0.001) ○ Hypocholesterolaemia <130 mg/dL (P <0.001) • The median survival time of 27 days (95% CI 19–35) was significantly longer than that of 14 days (95% CI 10–18) in the elevated serum LDH (≥313IU/L) group (HR 2.235, p<0.001). • *By multivariate analysis (Cox)*, *the following variables were independent and significant prognostic factors of poor survival time*: ○ ECOG PS ○ Medium–high level of pain ○ Fatigue ○ Hypotension ○ Elevated serum LDH (HR 2.087, p = 0.002) ○ Elevated serum CRP (HR 1.984, p = 0.002) ○ Elevated serum urate (HR 2.853, p <0.001) • *Statistically non significant*: ○ Haemoglobin ○ Pleural effusion ○ Pneumonia • A new scoring system was developed to estimate the survival time of terminally ill cancer patients using the 7 variables identified by multivariate analysis. The accuracy of prediction for study subjects (n = 93) is below: ○ *Prediction <3 weeks*: sensitivity 76%, specificity 67%, PPV 74%, NPV 70% ○ *Prediction <4 weeks*: sensitivity 71%, specificity 73%, PPV 85%, NPV 55% • Average serum LDH concentration was measured consecutively at 2 weeks and 1 week before death (n = 25) and was 5.04.04 +/- 347.11IU/L and 630.40 +/- 417.32IU/L respectively (p = 0.008).
Alsirafy SA *et al*. 2009, Saudi Arabia [30]	Retrospective cohort study To assess the predictive significance of abnormalities in serum sodium, potassium, calcium, magnesium and phosphate on the admission outcome and survival of patients with cancer referred to palliative care service.	Hospital inpatients referred to the palliative care team Single centre n = 259	Advanced cancer Median age: 52 years (range 14–87)	Median survival: 38 days	Retrospective Aetiology of electrolyte abnormalities not addressed	Level 2++ *Hawker et al*: 31/36 *Maltoni et al*: 6/7	• Hyponatremia was the most common electrolyte abnormality (64%). • *By univariate analysis*, *the following variables were significant prognostic factors of poor survival time*: ○ Serum sodium (p = 0.0008) ○ Serum calcium (p = 0.0008) ○ Serum magnesium (p = 0.0001) • Abnormalities in serum potassium and phosphate had no significant impact on admission outcome or overall survival. • The 3 electrolyte abnormalities associated with the highest inpatient death rate and shortest median survival were: ○ *Hypercalcaemia* (inpatient death rate: 69%, median survival: 12 days) ○ *Hypernatremia* (68%, 8 days) ○ *Hypermagnesemia* (62%, 12 days) • Combining these abnormalities in a simple scoring system divided the patients into 3 groups with significant difference in overall survival: ○ *Two abnormalities*: 8 days (95% CI 7–9) ○ *One abnormality*: 24 days (95% CI 10–38) ○ *No abnormalities*: 60 days (95% CI 26–94)
Hyodo, I *et al*. 2010, Japan [31]	Prospective cohort study (explorative) To develop a new prediction tool, JPOS-PI for terminally ill cancer patients with advanced solid tumors and to compare the new tool with PaP score and PPI.	• Inpatients treated in a hospice or hospital palliative care units across multiple centres in Japan • *Development sample*: ○ n = 201 ○ Median age: 63 years (SD 12) • *Test sample*: ○ n = 208 ○ Median age: 65 years (SD 12)	Terminally ill Advanced solid organ tumours Median age: 65 (SD 12 years)	• Median survival: ○ *Development sample*: 30 days ○ *Test sample*: 27 days	The accuracy and practical value of CPS remains controversial Ratio between the number of events (death) and the number of potential predictors <10 Requires invasive procedure Requires external validation	Level 2++ *Hawker et al*: 33/36 *Morita et al*: 5/7	• *Multivariate analysis (Cox) identified 5 significant predictors (p < 0*.*05) in the development sample*, *including*: ○ CPS ○ Disturbance in consciousness ○ Pleural effusion ○ WBC count ○ Lymphocyte percentage • JPOS-PI was developed using these predictors and divided patients into 3 risk groups. Median survival for each group was as follows: ○ *Group A*, *low risk*: 51 days ○ *Group B*, *intermediate risk*: 35 days ○ *Group C*, *high risk*: 16 days • Survival probability for more than 30 days in the development sample was as follows: ○ *Group A*, *low risk*: 78% ○ *Group B*, *intermediate risk*: 61% ○ *Group C*, *high risk*: 16% • JPOS-PI was studied in subsequent test sample, and constant results were obtained. • The 3 risk groups judged with JPOS-PI and PaP scores showed very similar survival in the test sample.
Tarumi, Y *et al*. 2011, Canada [32]	Prospective cohort study (confirmative) External validation of the PaP Score in hospital inpatients referred to the palliative care service in a large acute hospital in Canada.	Hospital inpatients referred to palliative care service in a single centre in Canada n = 958	Cancer, n = 777 (81.1%) Non-cancer, n = 181 (18.9%) Median age: 73 years	Median survival: 35 days (95% CI 31–39)	Includes cancer and non-cancer patients Variations in clinicians’ experience in making the predictions Until the PaP is tested in all hospitalized patients or all cancer patients, application of the Survival Rate by PaP Score requires caution Single centre Earlier published study with similar objectives [28]	Level 2++ *Hawker et al*: 35/36 *Maltoni et al*: 6/7	• The PaP Score subdivides the population into 3 groups with a different probability of survival at 30 days: ○ *Group A*: >70% ○ *Group B*: 30–70% ○ *Group C*: <30% [21] • This study externally validates the PaP Score in patients referred to the hospital palliative care team. The 3 groups, divided based on different ranges of PaP Score, had significantly different survival curves with 30-day-survival rates as follows: ○ *Group A*: 78% ○ *Group B*: 55% ○ *Group C*: 11% • Median survivals for the three groups were: ○ *Group A*: 89 days (95% CI 72–106) ○ *Group B*: 35 days (95% CI 30–40) ○ *Group C*: 4 days (95%CI 3–5)
Feliu, J *et al*. 2011, Spain [33]	Prospective cohort study (explorative) To identify more relevant clinical and laboratory variables that predict survival in terminally ill cancer patients, and to develop and validate a nomogram that predicts survival at 15, 30, and 60 days.	• Hospital oncology and palliative care unit inpatients and outpatients • n = 880: ○ *Training set*, n = 406 ○ *Validation set*, n = 474	• Various malignancies • Terminally ill • Median age: ○ *Training set*: 66.4 years (range 18–95) ○ *Validation set*: 67.2 years (range 17–96)	• Expected survival <6 months • Median survival: ○ *Training set*: 29.1 days ○ *Validation set*: 18.3 days	Some relevant survival parameters such as CPS, CRP, and comorbid conditions were not assessed in this study but might provide additional prognostic information Requires invasive procedure	Level 2++ *Hawker et al*: 35/36 *Maltoni et al*: 6/7	• *In univariate survival analysis (Cox)*, *a number of factors were statistically significantly associated with survival time including*: ○ Serum creatinine (p = 0.039) ○ Serum corrected calcium (p = 0.007) ○ Haemoglobin (p = 0.006) ○ Lymphocyte count (p = 0.000) ○ Serum bilirubin (p = 0.001) ○ Serum LDH (p = 0.000) ○ Serum cholesterol (p = 0.009) ○ Serum albumin (p = 0.000) • *Multivariate analysis (Cox) identified 5 variables independently prognostic*: ○ ECOG PS ○ TTD, a subjective parameter which may relate to tumour aggressiveness ○ Serum albumin (HR 0.729, 95% CI 0.621–0.856, p = 0.000) ○ Serum LDH (HR 1.127, 95% CI 1.058–1.200, p = 0.000) ○ Lymphocyte number (HR 0.876, 95% CI 0.791–0.970, p = 0.011) • *Statistically non significant factors by univariate analysis*: ○ Serum potassium ○ Serum sodium ○ GGT ○ ALP ○ Neutrophil count ○ Uric acid • A nomogram for predicting the probability of survival at 15, 30, and 60 days was constructed using the 5 variables identified by multivariate analysis. • Cumulative survival could be grouped into quartiles according to the score generated by the nomogram, and median survival by group was: ○ *Quartile 1*, *score ≤-0*.*45*: 83 days (95% CI 64–102) ○ *Quartile 2*, *score -0*.*45–0*: 33 days (95% CI 22–44) ○ *Quartile 3*, *score 0–0*.*34*: 24 days (95%CI 20–28) ○ *Quartile 4*, *score >0*.*34*: 10 days (95%CI 8–12) • The nomogram was validated with an external dataset that included 474 patients. The nomogram provided better AUC values than the PaP Score at 15, 30 and 60 days.
Gwilliam, B *et al*. 2011, UK [34]	Prospective cohort study (explorative) Development and validation of 4 prognostic models for predicting survival.	18 hospital, hospice and community palliative care services across the UK n = 1080	Advanced cancer Palliative	Median survival: 34 days	Models require external validation PiPS-B models requires invasive procedure	Level 2++ *Hawker et al*: 36/36 *Maltoni et al*: 6/7	• *On multivariate analysis*, *a number of variables independently predicted both two week and two month survival including*: ○ Serum CRP ○ WBC count ○ Platelet count ○ Urea • *A number of variables had prognostic significance only for 2 week including*: ○ Serum ALT level • *A number of variables had prognostic significance only for 2 month survival including*: ○ Lymphocyte count ○ Neutrophil count ○ Serum ALP level ○ Serum albumin • Separate prognostic models were created for patients without (PiPS-A) or with (PiPS-B) blood results. The AUC for all 4 models varied between 0.79–0.86. • PiPS-A scores can correctly order survival times for pairs of participants 68.9% of time. • PiPS-B scores can correctly order survival times for pairs of participants 67.5% of time. • The PiPS prognostic models can predict whether patients will survive for “*days*,” “*weeks*,” or “*months*”.
Cui, J *et al*. 2014, China [35]	Prospective cohort study (explorative) To develop a new prognostic scale to predict survival time of advanced cancer patients in China.	Hospital inpatients across 12 centres in China n = 320	Advanced cancer Expected to be in the last 6 months of life	Median survival: 34.5 days	Serum Na, total bilirubin, direct bilirubin, AST and ALP have not previously been included in other prognostic models. Their significance in survival prediction warrants further study Ratio between the number of events (death) and the number of potential predictors <10 Requires invasive procedure	Level 2+ *Hawker et al*: 32/36 *Maltoni et al*: 5/7	• *By univariate and multivariate analysis*, *14 variables were significantly associated with survival time including* ○ KPS ○ Pain ○ Ascites ○ Hydrothorax ○ Oedema ○ Delirium ○ Cachexia ○ WBC ○ Hemoglobin ○ Serum sodium ○ Total bilirubin ○ Direct bilirubin ○ AST ○ ALP • The new-ChPS Scale developed using the above prognostic factors. • When the prognostic score of a patient was > 12, the prediction of survival appeared to be < 7 days. • When the prognostic score of a patient was < 6, the prediction of survival appeared to be > 180 days (6 months). • *Statistically non significant factors by univariate analysis*: ○ Platelet count ○ Serum creatinine ○ Serum chlorine ○ Serum globulin
Kim, ES *et al*. 2014, Korea [36]	Prospective cohort study (confirmative) To validate the PiPS model for terminal cancer patients in Korea, and evaluate its value in clinical practice.	Hospital inpatients on a palliative care ward in a single centre in Korea n = 202	Advanced cancer Various malignancies Mean age: 62.6 years (SD 12.1)	Median survival: 25 days (95% CI 19–30)	Includes only hospital inpatients on palliative care ward. Further research is needed in order to generalise the findings in various clinical settings PiPs-B model requires invasive procedure Ratio between the number of events (death) and the number of potential predictors <10	Level 2++ *Hawker et al*: 35/36 *Maltoni et al*: 5/7	Gwilliam *et al*. developed the PiPS prognostic models, which can be used without (PiPS-A) or with (PiPS-B) blood results, and can predict whether patients are in the last “*days*,” “*weeks*,” or “*months*” of life [34]. In this study, the PiPs models are externally validated in patients admitted to a palliative care ward in Korea. The overall accuracy between the PiPS-A and actual survival was 52.0%. The overall accuracy between the PiPS-B and actual survival was 49.5%. The overall accuracy between clinicians’ estimates and actual survival was 46.5%, which were lower than those of the PiPS models. The “*weeks*” and “*months*” groups showed significantly prolonged survival rates than “*days*” group did in both PiPS-A and PiPS-B, by the Kaplan-Meier method. The sensitivity, specificity, PPV, and NPV of predictions of survival using the PiPS-A and the PiPS-B predictor models were variable. In the “*days*” and “*weeks*” groups, the PiPS-A/PiPS-B predictions and clinicians’ estimates were consistent with actual median survival.
Amano, K *et al*. 2015, Japan [37]	Prospective cohort study (explorative) To investigate CRP as a prognostic marker in advanced cancer.	Patients receiving inpatient or community palliative care services across 58 centres in Japan n = 1511	• Advanced cancer: ○ *Locally extensiv*e: n = 278 ○ *Metastatic*: n = 1233 • Various malignancies including haematological • Mean age: ○ *Low*, *CRP <1 mg/dL*: 68.8 years (SD 13.4) ○ *Moderate*, *CRP ≥1 - <5 mg/dL*: 69.1 years (SD 12.1) ○ *High*, *CRP ≥5 - <10 mg/dL*: 68.4 years (SD 12.6) ○ *Very high*, *CRP ≥10 mg/dL*: 66.3 years (SD 13.4)	• Median survival: ○ *Low*, *CRP <1 mg/dL*: 72.0 days (95% CI 58–86) ○ *Moderate*, *CRP ≥1 - <5 mg/dL*: 42.0 days (95% CI 36–48) ○ *High*, *CRP ≥5 - <10 mg/dL*: 25.0 days (95% CI 22–28) ○ *Very high*, *CRP ≥10 mg/dL*: 17.0 days (95% CI 14–20)	Does not distinguish between different malignancies which may influence serum CRP levels No data on acute infections, acute medical conditions and cachexia, which may influence serum CRP levels Requires invasive procedure	Level 2++ *Hawker et al*: 36/36 *Maltoni et al*: 6/7	• Survival rate decreased and mortality rate increased with increasing CRP level. • Patients classified into 4 groups based on CRP level. Median survival times were: ○ *Low*, *CRP <1 mg/dL*: 72.0 days (95% CI 58–86) ○ *Moderate*, *CRP ≥1 - <5 mg/dL*: 42.0 days (95% CI 36–48) ○ *High*, *CRP ≥5 - <10 mg/dL*: 25.0 days (95% CI 22–28) ○ *Very high*, *CRP ≥10 mg/dL*: 17.0 days (95% CI 14–20) (*p* < 0.001) • The differences in survival and 30-, 60- and 90-day mortality rates among the groups were statistically significant (p < 0.001). • In the multivariate adjusted model (Cox), a significantly higher risk of mortality was observed in the moderate-, high- and very high-CRP groups than in the low-CRP group: ○ *Moderate*, *CRP ≥1 - <5 mg/dL*: HR 1.47 (95% CI 1.24–1.73), p < 0.001 ○ *High*, *CRP ≥5 - <10 mg/dL*: HR 2.09 (95% CI 1.74–2.50), p < 0.001 ○ *Very High*, *CRP ≥10 mg/dL*: HR 2.55 (95% CI 2.13–3.05), p < 0.001
Baba, M *et al*. 2015, Japan [38]	Prospective cohort study (confirmative) Independent validation of the modified PiPS predictor models in 3 palliative care settings in Japan.	• 3 palliative care settings in Japan: ○ Hospital inpatients ○ Palliative care inpatients ○ Community palliative care services • *Modified PiPs A model*: n = 2212 • *Modified PiPs B model*: n = 1257	• Advanced cancer • Under hospital or community palliative care services • Mean age: ○ *Hospital inpatients*: 65.5 years (SD 12.6) ○ *Palliative care unit inpatients*: 70.4 years (SD 12.3) ○ *Community palliative care service*: 73.0 years (SD 12.5)	• Median survival: ○ *Hospital inpatients*: 45 days ○ *Palliative care unit inpatients*: 24 days ○ *Community palliative care service*: 37 days	Includes patients receiving chemotherapy (n = 359), however subgroup analysis of patients not receiving chemotherapy demonstrated the same conclusions Modified PiPs model not compared to CPS or another prognostic model Ratio between the number of events (death) and the number of potential predictors <10	Level 2++ *Hawker et al*: 35/36 *Maltoni et al*: 6/7	• Gwilliam *et al*. developed the PiPS prognostic models, which can be used without (PiPS-A) or with (PiPS-B) blood results, and can predict whether patients are in the last “*days*,” “*weeks*,” or “*months*” of life [34]. • In this study, the original PiPS models were modified so that ratings could be obtained without patient participation, and externally validated across all palliative care settings. • In all palliative care settings, both the modified PiPS-A and PiPS-B was successfully validated and identified 3 risk groups with different survival rates (p < 0.001). • The median survival for each group classified by PiPs-A was as follows: ○ *Group A*, *days*: 10 days (95% CI 8–12) ○ *Group B*, *weeks*: 39 days (95% CI 34–44) ○ *Group C*, *months*: 134 days (95% CI: 108–160) • The absolute agreement ranged from 56–60% in the PiPS-A model and 60–62% in the PiPS-B model, similar to previous reports.
Baba, M *et al*. 2015, Japan [39]	Prospective cohort study (confirmative) To investigate the feasibility and accuracy of the PaP Score, D-PaP Score, PPI and modified PiPS models across 58 palliative care services in Japan.	• 4 palliative care settings across 58 centres in Japan: ○ Hospital inpatients (n = 554) ○ Palliative care units inpatients (n = 820) ○ Community palliative care services (n = 472) ○ Palliative chemotherapy (n = 515) • n = 2361	• Advanced cancer: ○ *Locally extensive*: n = 485 ○ *Metastatic*: n = 1876 • Various malignancies including haematological • Under hospital or community palliative care services • Mean age: ○ *Hospital inpatients*: 68.1 years (SD 12.7) ○ *Palliative care unit inpatients*: 70.8 years (SD 12.2) ○ *Community palliative care service*: 74.3 years (SD 11.9) ○ *Palliative chemotherapy*: 62.8 years (SD 12.0)	• Median survival: ○ *Hospital inpatients*: 37 days (95% CI 31.6–42.4) ○ *Palliative care unit inpatients*: 25 days (95% CI 22.6–27.4) ○ *Community palliative care services*: 37 days (95% CI 32.1–41.9) ○ *Palliative chemotherapy*: 48 days (95% CI 41.9–54.1)	Physicians not blinded among ratings of each prognostic score Direct statistical comparison not possible	Level 2++ *Hawker et al*: 35/36 *Maltoni et al*: 6/7	The D-PaP Score integrates delirium as an additional predictor to the PaP score. In this study, the feasibility of previously described predictor models: PaP Score, D-PaP Score, PPI, and modified PiPS models were assessed across all palliative care settings. The feasibility of PPI and modified PiPS-A was >90% in all groups, followed by PaP and D-PaP scores; modified PiPS-B had the lowest feasibility. The accuracy of prognostic scores was ≥69% in all groups and the difference was within 13%, while c-statistics were significantly lower with the PPI than PaP and D-PaP scores. All prognostic tools can predict short (≤13 days or ≤21 days) and long (≥30 days, ≥42 days, or ≥56 days) survival probability in all these groups using the cut-off points proposed in the original studies.
Malik S *et al*. 2015, Australia [40]	Retrospective cohort study To determine the reversibility of hypercalcaemia amongst patients whose underlying with intravenous bisphosphonates, and assess the prognostic value of correction in serum Ca.	Single centre Palliative care unit in Australia n = 66	Hypercalcaemia treated with IV bisphosphonate Cancer Median age: 72 years	• Median survival: ○ *Responders*: 22 days ○ *Non-responders*: 3 days	Serum PTH not routinely measured Other causes of hypercalcaemia could not be excluded Retrospective Small sample size	Level 2++ *Hawker et al*: 33/36 *Maltoni et al*: 6/7	A reduction in serum calcium concentration was associated with a significantly prolonged survival, as well as symptomatic improvement, irrespective of whether normocalcaemia was achieved. Pre-treatment serum calcium level did not independently effect survival, in contrast to the literature.
Taylor, P *et al*. 2015. UK [41]	Retrospective cohort study (investigative) Measurement of blood variables over 3 months of data collection in terminally ill hospital patients with solid organ malignancies.	Hospital inpatients in a single tertiary centre n = 102	Terminally ill Solid organ malignancies Median age: 73 years (range 39–102)	Last 2 weeks of life	No data collected on hydration status, clinically assisted hydration, oxygen therapy or presence of acute illness Retrospective Reliance of death certificate for cause of death Ratio between the number of events (death) and the number of potential predictors <10	Level 2++ *Hawker et al*: 36/36 *Maltoni et al*: 4/7	Heart rate significantly increased during the last week of life, although the model intercept value only reached 99bpm on the day of death. Changes were not observed in blood pressure, and intercept values remained clinically unremarkable All respiratory variables measured changed significantly over the final 2 weeks of life with a rise in respiratory rate and reduction in oxygen saturation. Serum urea and creatinine showed a clinically and statistically significant increase. Serum sodium showed a statistically significant but clinically insignificant increase, while serum potassium showed no significant change over time. Evidence of leucocytosis and reduced lymphocyte counts, but there was no clinically significant change in the last 2 weeks of life. Recorded values were abnormal indicating that these variables are more likely to change slowly over weeks rather than days. Serum albumin showed a significant change over time and abnormal values as death approaches, showing prognostic power over both longer and shorter timescales. Other long-term variables including haemoglobin showed a significant change over 3 months of data collection, but results were masked by red cell transfusion.
Yoon, J *et al*. 2015, Korea [42]	Retrospective cohort study (explorative) To analyse the relationship between serum sodium and survival in patients with terminal cancer.	Tertiary hospital palliative care unit in Korea n = 576	• Terminal cancer: ○ *Locally extensive*: n = 80 ○ *Metastasis*: n = 496 • Various carcinomata • Mean age: 62 years (SD 13.14)	Median survival: 15 days	Single centre Retrospective Did not assess hydration, use of diuretics or clinically assisted hydration Did not assess changes in serum Na level over time Requires invasive procedure	Level 2+ *Hawker et al*: 35/36 *Maltoni et al*: 5/7	• In univariate analysis (Cox), CRP showed significant association with survival (HR 1.22, p<0.001). • In univariate analysis (Cox), hyponatraemia ≤ 125mEq/L was significantly associated with poor prognosis compared with eunatraemia (HR 1.91, p<0.001). • In multivariate analysis (Cox), CRP showed a significant association with survival (HR 1.16, p< 0.001). • In multivariate analysis (Cox), hyponatraemia showed statistical significance regarding survival time compared with eunatraemia: ○ *Serum Na ≤125mEq/L*: HR 1.75, p<0.001 ○ *Serum Na 126 – 135mEq/L*: HR 1.19, p = 0.048 ○ Serum Na ≤125mEq/L showed the shortest survival time compared with eunatraemia • Hyponatraemia is an independent prognostic marker in advanced cancer patients.
Miura, T *et al*. 2015, Japan [43]	Prospective cohort study (explorative) To investigate the correlation between GPS and survival among cancer patients in all palliative settings in Japan.	All palliative care settings across 58 centres in Japan n = 1160	Advanced care Various malignancies Under palliative care service Median age: 72 years (range 63–80)	• Median survival: ○ *GPS 0*: 58 days (95% CI 48–81) ○ *GPS 1*: 43 days (95% CI 37–50) ○ *GPS 3*: 21 days (95% CI 19–24)	No laboratory data for 1263 (52.1%) of participants and excluded from analysis Requires invasive procedure	Level 2++ *Hawker et al*: 35/36 *Maltoni et al*: 6/7	• The GPS is a simple inflammation-based prognostic score that represents elevated CRP levels (>10 mg/L) and hypoalbuminaemia (<35 g/dL). The GPS is scored as 0, 1 or 2. • In this study, the prevalence of GPS 2 was 70.9%. • The sensitivity and specificity for 3-week prognosis of GPS 2 were 0.879 and 0.410 respectively • *Median survival time based on GPS was as follows (p<0*.*001)*: ○ *GPS 0*: 58 days (95% CI 48–81) ○ *GPS 1*: 43 days (95% CI 37–50) ○ *GPS 3*: 21 days (95% CI 19–24) • The NLR is the neutrophil-lymphocyte ratio, and a score ≥ 4 has been shown to be the best cut of value for predicting prognosis. • *Univariate analysis (Cox) showed a significant association with survival and the following variables*: ○ GPS of 1 or 2 ○ NLR ≥4 • Multivariate analysis (Cox) showed that NLR ≥ 4 was an independent prognostic factor (HR 1.43, 95% CI, 1.17–1.75 p < 0.001). • The GPS is a good prognostic indicator for cancer patients across all palliative care settings.
Coyle S *et al*. 2016, UK [44]	Prospective cohort study (investigative) To collect urine samples from patients towards the end of life and analyse them for VOCs using GC-MS, and water-soluble metabolites using NMR.	Hospice inpatients Advanced cancer, n = 8 Non-cancer, n = 1 Healthy controls, n = 4	Advanced cancer Expected prognosis < 4 weeks	Median survival: 25 days	Small sample size Single centre Includes non cancer patients	Conference abstract Level: N/A *Hawker et al*: N/A *Maltoni et al*: N/A	GCMS analysis identified over 390 VOCs. There were 279 VOCs in normal urine. The number of statistically significant VOCs increased from 12 at 4 weeks before death, to 43 at 3–5 days before death. A trend analysis for the individual VOCs showed that of these VOCs, 1 was significant at four weeks before death and 22 VOCs were significant at 3–5 days before dying. The steepest rise in significant VOCs was seen in the last week of life. These VOCs may predict the dying process.
Niki K *et al*. 2016, Japan [45]	Retrospective cohort study (explorative) To identify short-term prognostic variables by detecting a statistical change-point in laboratory test values. To compare the WPCBAL and WPBAL scores to the GPS score.	University hospital inpatients n = 121	Advanced cancer Mean age: 58.1 years (SD 17.0)	Last 2 weeks of life	Single centre Requires external validation Requires invasive procedure	Conference abstract Level: N/A *Hawker et al*: N/A *Maltoni et al*: N/A	The GPS is a simple inflammation-based prognostic score that represents elevated CRP levels (>10 mg/L) and hypoalbuminaemia (<35 g/dL). The GPS is scored as 0, 1 or 2. The WPCBAL score was derived from the WBC, platelet count, BUN, AST, LDH, and CRP levels. The WPBAL score was derived from the WBC, platelet count, BUN, AST, and LDH levels. The WPCBAL and WPBAL scores showed a higher sensitivity, specificity, negative predictive value, and relative risk for the prognosis within 2 weeks of death compared to those of the GPS considering a less than three weeks prognosis. While the WPCBAL and WPBAL scores showed an equivalent positive predictive value to the GPS, both positive and negative likelihood ratio were superior to those of GPS.
Paulsen O *et al*. 2016, Norway [46]	Randomised controlled trial Post hoc analysis to investigate whether inflammatory markers at baseline (protein production in the serum detected by Multiplex technology) were associated with symptom intensity; or associated with response to corticosteroid therapy.	Palliative care community and inpatients at 5 centres in Norway n = 49	Advanced cancer Opioid therapy	Median survival: 86 days	Only pre-treatment samples available for analysis No direct comparison with corticosteroid group Small sample number 6 patients excluded from analysis due to missing data Protein and not RNA levels detected Requires invasive procedure Prognostic significance not assessed	Conference abstract Level: N/A *Hawker et al*: N/A *Maltoni et al*: N/A	• Cytokines IL-2, IL-4, IL-8, IL-10, IL-12(p70), TNF-α, IFN-γ, and MIP-1α values were below the lower limit of detection in the serum in more than 37 of the 43 analyzed patients, and these data were excluded from the analyses. • The following cytokines were elevated above the lower limit of detection in the serum: ○ sTNF- r1 (n = 43, median concentration 10917 pg/mL) ○ MCP-1 (n = 43, median 64.1 pg/mL) ○ IL-18 (n = 43, median 103.2 pg/mL) ○ TGF-β1 (n = 43, median 45145 pg/mL) ○ MIF (n = 42, median 134.9 pg/mL) ○ IL-6 (n = 21, median 2.33 pg/mL) ○ IL-1ra (n = 11, median 21.7 pg/mL) ○ IL-1β (median 0.15 pg/mL) ○ CRP (median 44 pg/mL) ○ ESR (median 42 pg/mL) • EORTC QLQ-C30 physical and role function (markers of quality of life) were highly correlated to CRP and sTNF-r1, and IL-6 and ESR, respectively. • *Medium correlations were found between*: ○ Appetite and CRP, IL-6 and IL-1β ○ Fatigue and IL-1ra ○ Cognitive function and TGF-β1 ○ Dyspnoea and IL-1β.
Wrafter S *et al*. 2016, Ireland [47]	Cohort study (confirmatory) To assess the prognostic value of CRP, albumin and CRP-based scores (CAR, mGPS with standard and low cut-offs) in cancer admissions to a specialist palliative care unit in Ireland.	Specialist palliative care unit inpatients in a single centre n = 120	Advanced cancer	• Mean survival: ○ *High and normal CRP*: 80 versus 29 days; p< 0.001 ○ *Low and normal albumin*: 35 versus 87 days; p< 0.01		Conference abstract Level: N/A *Hawker et al*: N/A *Maltoni et al*: N/A	85% (n = 94) had serum CRP > 10mg/L. 96% (n = 106) had serum albumin <35g/L. There was a significant mean survival difference between high and normal CRP (80 versus 29 days; p< 0.001) and low and normal albumin (35 versus 87 days; p< 0.01). CAR, the CRP/albumin ratio is an inflammatory prognostic model that has been shown to predict survival in various malignancies. The mGPS combines serum albumin and CRP into a risk stratification score, and has been shown to predict survival in various malignancies. The mGPS divides patients into 3 subgroups: 0, 1 and 2. Neither of these predictive models has been used in palliative care settings. CAR was above published cut-offs for 84% (n = 92). A significant survival difference was found between mGPS groups at 7(p = 0.01) and 180 (p = 0.02) but not 30 days. A lower CRP threshold did not improve prognostication.

Only prognostic factors relevant to the objectives of this review are presented here.

AGP = α1-acid glycoprotein; ALP = alkaline phosphatase; ALT = alanine aminotransferase; AST = aspartate transaminase; AUC = area under the curve; BCI = B12 CRP index; BUN = blood urea nitrogen; B12 = vitamin B12; CACS = cancer anorexia-cachexia syndrome; CAR = CRP/albumin ratio; CPS = Clinicians’ Prediction of Survival; CRP = C-reactive protein; D-PAP = Delirium-Palliative Prognostic Score; ECOG PS = Eastern Cooperative Oncology Group Performance Status; ESR = erythrocyte sedimentation rate; GC-MS = Gas Chromatography Mass Spectrometry; GGT = gamma-glutamyl transpeptidase; GPS = Glasgow Prognostic Score; IFNγ = interferon gamma; IL-1ra = interleukin-1 receptor antagonist; IL-1β = interleurkin-1 beta; IL-2 = interleukin-2; IL-4 = interleukin-4; IL-6 = interleukin-6; IL-8 = interleukin-8; IL-10 = interleukin-10; IL-12(p70) = interleukin-12(p70); IL-18 = interleukin-18; INR = international normalised ratio; JPOS-PI = Japan Palliative Oncology Study-Prognostic Index; KPS = Karnofsky Performance Status; LDH = lactate dehydrogenase; LIF = leukaemia inhibiting factor; MCP-1 = monocyte chemoattractant protein-1; m-GPS = modified Glasgow Prognostic Score; MIF = macrophage migration inhibitory factor; MIP-1α = macrophage inflammatory protein-1α; MSSE = mini mental state examination; new-ChPS = new-Chinese Advanced Cancer Patients Scale; NLR = neutrophil-lymphocyte ratio; NMR = Nuclear Magnetic Resonance Spectrometry; NPV = negative predictive value; PaP Score = Palliative Prognostic Score; PINI = Prognostic Inflammatory and Nutritional Index; PiPS = Prognosis in Palliative Care Study; PiPS-A = modified Prognosis in Palliative Care Study-A; PiPS-B = modified Prognosis in Palliative Care Study-B; PPI = Palliative Performance Index; PPV = positive predictive value; PTH = parathyroid hormone; PTHrP = parathyroid hormone related protein; RNA = ribonucleic acid; sTNF- r1 = soluble tumour necrosis factor receptor 1; TGF-β1 = transforming growth factor β1; TNFα = tumour necrosis factor alpha; TTD = time from initial diagnosis to diagnosis of terminal disease; VOCs = volatile organic compounds; WBC = white blood cell count; WPBAL = WBC/platelet/BUN/AST/LDH prognostic score; WPCBAL = WBC/platelet/CRP/BUN/AST/LDH prognostic score.

### Study characteristics

The characteristics of the included studies are summarised in Table 2. A formal meta-analysis was not conducted because of the heterogeneity of the published studies. A number of studies utilised univariate rather than multivariate analysis. Both types of studies were included to ensure a comprehensive summary of the literature. The use of the Evidence Based Medicine modified GRADE system has highlighted the limitations of univariate analysis for these types of studies [17].

Table 3 subdivides prognostic biomarkers by grades of evidence. Only five articles investigated changes in concentrations of identified biomarkers in the last days to weeks of life (Table 3).

**Table 3:** Prognostic biomarkers subdivided by evidence based medicine modified GRADE criteria.

GRADE	Quality of Evidence	Biomarker
A	High	Serum CRP^a^ Serum WBC^a^ Serum lymphocyte count Serum albumin Serum ALP Serum urea/BUN^a^ Serum Na^a^
B	Moderate	Platelet count^a^ Serum prealbumin Serum vitamin B12 Serum bilirubin Serum AST Serum ALT^a^ INR Serum LDH^a^ Serum cholesterol Serum urate^a^ Serum pseudocholinesterase
C	Low	Plasma IL-6^a^^b^ Plasma sTNF- r1^b^ Plasma MCP-1^b^ Plasma IL-18^b^ Plasma TGF-β1^b^ Plasma MIF^b^ Plasma IL-1ra^b^ Plasma IL-1β^b^ Plasma IL-6^b^ ESR^b^ Serum haemoglobin Serum GGT Serum creatinine Serum calcium Serum magnesium Plasma glucose Urine VOCs^b^
**D**	Very Low	Proteinuria Serum potassium Serum phosphate

ALP = alkaline phosphatase; ALT = alanine aminotransferase; AST = aspartate transaminase; BUN = blood urea nitrogen; CRP = C-reactive protein; IL-1ra = interleukin-1 receptor antagonist; IL-1β = interleukin-1 beta; IL-6 = interleukin-6; IL-18 = interleukin-18; ESR = erythrocyte sedimentation rate; GGT = gamma-glutamyl transferase; INR = international normalised ratio; LDH = lactate dehydrogenase; MCP-1 = monocyte chemoattractant protein-1; MIF = macrophage migration inhibitory factor; sTNF- r1 = soluble tumour necrosis factor receptor 1; TGF-β1 = transforming growth factor β1; VOCs = volatile organic compounds; WBC = white blood cell count.

^a^Biomarkers detectable in the blood in the last two weeks of life

^b^Prognostic significance not assessed

The methodological quality of the included studies ranged between 26–36 using the Hawker *et al*. appraisal tool [16]. Using the seven-point checklist of quality criteria, the selected studies ranged between 3–6 [8]. Spearman’s rank correlation coefficient demonstrated a statistically significant rank correlation between assessors (rs 1⁄4 0.955 p = 0.000 and rs 1⁄4 0.921 p = 0.000, respectively). Frequently, sample size calculations were not conducted, and some studies lacked information about the study setting and patient characteristics. Further, the majority (97%) of studies had convenience rather than random sampling, a reliable method for identification of the date of death was often poor, and a large number of potential prognostic factors were analysed despite small sample sizes, which meant that the ratio between the number of deaths and the number of potential prognostic factors frequently scored less than ten.

## Discussion

This is the first systematically structured review to evaluate prognostic biomarkers in a heterogeneous group of patients with advanced cancer. Seven prognostic biological factors had *Grade A* evidence: lymphocyte count, white blood cell (WBC) count, serum albumin, sodium, C-reactive protein (CRP), urea and alkaline phosphatase (ALP) (Table 3). Few studies have specifically investigated changes in biomarkers in the last days to weeks of life. In the last two weeks of life a number of biomarkers were elevated in the blood including: WBC count, platelet count, serum CRP, urea, urate, alanine transaminase (ALT), lactate dehydrogenase (LDH), sodium and plasma interleukin-6 (IL-6). However limitations exist as only five studies specifically investigated serial measurements of candidate biomarkers in the last weeks of life.

A number of common themes emerged: systemic inflammation, haematological changes, CACS, hepatic dysfunction, renal dysfunction, and electrolyte changes. A number of biomarkers, such as serum albumin could be explained by multiple themes.

### Systemic inflammation & haematological changes

There is consistent evidence that the presence of a systemic inflammatory response is associated with reduced survival in patients with a variety of solid organ tumors [48–50]. Elevated serum CRP levels are associated with poor prognosis independent of tumour stage [37,51]. IL-6, interleukin-1 (IL-1) and tumour necrosis factor alpha (TNFα) induce the hepatic synthesis of CRP. Further, elevated CRP and IL-6 are associated with CACS in advanced cancer [51]. Measurement of serum CRP is readily available and is an ideal biomarker of systemic inflammation. There is *Grade A* evidence that elevated serum CRP is an independent prognostic factor in advanced cancer [18,22,29,34,37,42]. Amano *et al*. demonstrated a clear dose-related effect between elevated serum CRP and prognosis, and patients could be divided into four groups based on CRP concentrations [37] (Table 2).

Only two studies investigated the role of inflammatory cytokines at the end of life [25,46], this may be because most cytokines have a short plasma half-life and assays are expensive in relation to serum CRP. Iwase *et al*. measured changes in plasma levels of various cytokines in 28 terminally ill cancer patients with cachexia [25]. Only IL-6 was detected in all patients at a concentration greater than 10pg/mL [25]. The concentration of IL-6 was seen to gradually rise during the early stages of cachexia followed by a sharp rise in the week prior to death [25]. It was hypothesised that circulating macrophages and T lymphocytes produce IL-6 in response to tumour burden, or plasma IL-6 is produced by the tumour mass itself [25,52]. IL-6 is a pro-inflammatory cytokine that regulates immune reactions to tissue damage. There is a growing consensus that plasma IL-6 is a prognostic factor in solid organ malignancies [53,54], however changes in plasma IL-6 levels are affected by carcinoma type [55].

Paulsen *et al*. identified that a number of inflammatory cytokines were significantly elevated in the plasma in the last months of life [46] (Table 2). Interestingly, soluble tumour necrosis factor receptor-1 (sTNF-r1), IL-6, CRP and erythrocyte sedimentation rate (ESR) were highly correlated with quality of life, and interleukin-1β (IL-1β) was moderately correlated with breathlessness[46]. Further research is required to confirm these preliminary findings and assess their prognostic significance.

There is *Grade A* evidence that WBC count is an independent predictor of survival in advanced cancer [31,34,35]. In a UK multicentre cohort study, Gwilliam *et al*. demonstrated WBC count and platelet count independently predicted survival at two months and two weeks in a heterogeneous population of advanced cancer patients across a variety of palliative care settings [34].

There is *Grade A* evidence that lymphopenia is an independent predictor of survival in advanced cancer [23,31,33,34,41,43]. Gwilliam *et al*. confirmed that lymphocyte count was a significant predictor of two-month but not two-week survival [34]. The mechanisms driving lymphopenia in advanced cancer are unknown. Immunodeficiency in advanced cancer has been well documented and there is a significant trend of decreasing functional T cell populations (including CD4, CD8, CD4:CD8 ratio and naïve T cells) with cancer progression: this is associated with increased morbidity and mortality [56]. WBC differential rather than WBC counts may be a more useful prognostic marker for future studies.

The prognostic role of thrombocytopaenia (*Grade B*) [29,34] and haemoglobin count (*Grade C*) [33] has been indicated but further research is needed to confirm these findings.

### Cancer anorexia-cachexia syndrome

CACS is a significant prognostic factor in advanced cancer [8] and the biological mechanisms have been extensively studied [57,58]. There is *Grade A* evidence for the independent prognostic role of hypoalbuminaemia in advanced cancer [19,27,33,34,41]. It is hypothesised that hypoalbuminaemia is caused by a combination of hepatic dysfunction and CACS. Interestingly, Gwilliam *et al*. demonstrated hypoalbuminaemia was a predictor of two-month but not two-week survival, suggesting serum albumin is a predictor of dying over a longer timeframe [34]. This is contrary to the findings by Taylor *et al*. who demonstrated a significant change over time and as death approached [41]. These findings highlight the need for further prospective studies that analyse serial measurements of serum albumin at the end of life.

One article demonstrated low serum prealbumin as a significant predictor of survival in terminally ill cancer patients by multivariate analysis (*Grade B*) [24]. Albumin and prealbumin levels reflect the visceral protein pool [24]. Given that prealbumin has a shorter half-life than albumin; prealbumin may be a more sensitive marker of nutritional status [24].

### Hepatic dysfunction

A number of biomarkers of liver dysfunction have been indicated as prognostic in advanced cancer including: serum vitamin B12, albumin, bilirubin, aspartate aminotransferase (AST), ALT, ALP, gamma-glutamyl transferase (GGT), international normalised ratio (INR), LDH and cholesterol. The aetiology of hepatic dysfunction at the end of life is unclear. Certainly, studies have demonstrated mixed results on the prognostic significance of liver metastasis in advanced cancer [27,29,33,34,43].

There is *Grade B* evidence for the independent prognostic significance of elevated serum vitamin B12 in advanced cancer [18]. Geissbühler *et al*. demonstrated an inverse relationship between survival and serum vitamin B12 levels [18]. There was also a strong correlation between the presence of metastasis or hepatic dysfunction and an elevated serum vitamin B12 [18]. Importantly, there was no difference in serum vitamin B12 levels in patients with haematological malignancies (a potential confounding factor) compared to other cancers [18]. To our knowledge no additional studies have been published that investigate vitamin B12 as a prognostic factor.

Studies assessing serum bilirubin and other liver function tests have found mixed results. There is *Grade B* evidence that hyperbilirubinaemia is an independent prognostic factor [26,29,33,35]. Laboratory parameters ALP (*Grade A*), AST (*Grade B*) and ALT (*Grade B*) proved to be prognostically significant in at least one multivariate analysis [24,34,35]. Gwilliam *et al*. also found ALT predictive of two-week, but not two-month survival [34]. There are conflicting results whether GGT is prognostic (*Grade C*) [33,35]. These contrasting results may be explained by the geographical differences in patient groups, the type of patients included and differences in the methods of biochemical analysis of liver function tests. In contrast to serum AST, elevations in serum ALT are rarely seen in conditions other than liver parenchymal damage and may be a more sensitive marker for liver disease [24].

Prolonged INR was an independent prognostic factor by multivariate analysis in two prospective studies of terminally ill cancer patients (*Grade B*) [26,29]. To our knowledge, no studies have been published that measure serial changes in INR and liver function tests at the end of life.

Serum LDH is an independent prognostic factor in advanced cancer [26,29,33] (*Grade B*). Suh *et al*. demonstrated a significant increase in LDH concentrations in the last two weeks of life [29]. This retrospective study was limited however by sample size and was restricted to hospitalised patients [29]. Prospective multicentre studies across a variety of palliative care settings are required to confirm these preliminary findings. It has been extensively published that elevated serum LDH in cancer reflects tumour burden and aggressiveness [29,59]. Serum concentrations can be further elevated due to hepatic necrosis caused by hepatic dysfunction and/or metastasis in advanced cancer[29,60].

There is *Grade B* evidence for the prognostic role of hypocholesterolaemia in advanced cancer [26,29,33]. A number of risk factors for hypocholesterolaemia have been identified including: cancer, hepatic dysfunction, CACS, haematological disease and elderly populations [61].

### Renal dysfunction

A number of potential prognostic biomarkers of renal dysfunction have been indicated in advanced cancer. Maltoni *et al*. demonstrated insufficient evidence for the prognostic role of proteinuria in advanced cancer [8]. Proteinuria is prognostic in a number of solid organ tumours [62,63] however, to our knowledge, there have been no recent published studies that investigate proteinuria as a predictor of survival in advanced cancer.

Elevated serum urea was demonstrated as a significant predictor of survival in three studies by multivariate analysis (*Grade A*) [24,34,41]. Gwilliam *et al*. identified serum urea as an independent predictor of two-week and two-month survival in advanced cancer [34]. Taylor *et al*. also found that serum urea and creatinine showed a statistically and clinically significant increase in the last two weeks of life [41]. There is *Grade C* evidence that serum creatinine is prognostic in advanced cancer [26,33].

There is *Grade B* evidence that serum urate is a significant prognostic factor in advanced cancer [26,29,33] and serum urate levels were significantly increased in the last two weeks of life [26]. A number of potential explanations for raised serum urate concentrations have been hypothesised including: renal dysfunction and cellular injury caused by hypoxia and/or inflammation [26].

The mechanisms for renal dysfunction at the end of life are unclear. Oliguria was identified as a significant prognostic factor in two univariate analyses [26,29]. It is hypothesised that glomerular filtration rate is reduced at the end of life. Although hypotension is a prognostic factor, there are conflicting results whether blood pressure falls during the dying phase [41,64].

### Electrolyte changes

There is *Grade A* evidence that serum sodium is a significant predictor of survival in advanced cancer [30,35]. These studies involved hospitalised patients; and acute illness may be a confounding factor. One multivariate analysis identified hyponatraemia as an independent predictor of survival in advanced cancer [42]. The most common aetiology of hyponatraemia in cancer is syndrome of inappropriate secretion of antidiuretic hormone (SIADH) [42,65]. Conversely, hypernatraemia at the end life is commonly caused by dehydration [30] and is associated with shorter overall survival in hospitalised patients receiving palliative care [30]. Although, Taylor *et al*. demonstrated a statistically significant elevation in serum sodium in the last two weeks of life, results were not clinically significant [41]. Serum sodium levels rather than serum sodium may be a more useful prognostic factor for future studies.

The prognostic significance of hypercalcaemia has been described in a number of solid organ and haematological malignancies including: lung [66], prostate [67], renal [68], head and neck [69,70] and the aerodigestive tract [71]. There is conflicting evidence whether hypercalcaemia predicts survival in advanced cancer (*Grade C*). By univariate analysis, Alsirafy *et al*. found that hypercalcaemia was associated with a 69% inpatient death rate in hospitalised patients [30]. However, an additional three articles failed to demonstrate statistical significance [27,33,40]. Contrasting results may reflect differences in study settings, tumour type, and the presence of bony metastasis. One univariate analysis identified hypermagnesemia to be predictive of survival (*Grade C*) in patients referred to the hospital palliative care team [30].

Although Cui *et al*. demonstrated serum potassium as a prognostic factor by univariate analysis[35], an additional three articles were identified which failed to demonstrate statistical significance including two multivariate analyses (*Grade D*) [30,33,41]. In one univariate analysis, abnormal plasma glucose levels were predictive of survival (*Grade C*) [35].

### Non-invasive research methodologies

Coyle *et al*. recently presented preliminary results of a statistically significant increase in the number of volatile organic compounds (VOCs) in the urine in the last weeks of life using Gas Chromatography Mass Spectrometry (GC-MS) [44]. Interestingly, the steepest rise in significant VOCs was seen in the last week of life [44].

### Prognostic models

Based on the outcome of these studies a number of prognostic models have been developed to assist clinicians. Simmons *et al*. recently reviewed the role of prognostic models in advanced cancer [12]. They concluded that ‘various prognostic tools have been validated, but vary in their complexity, subjectivity and therefore clinical utility’ [12].

### What makes this study unique?

The Neuberger review made a number of recommendations to improve end of life care, including research into the biology of dying [1]. This is the first study to specifically investigate biomarkers in advanced cancer patients in the last months of life and attempts to extrapolate which biological processes are affected. A number of common themes emerged including: systemic inflammation, organ dysfunction and CACS.

### What is the significance of the findings of this analysis?

This review demonstrates that there are many biological prognostic factors that have an association with the dying process. Although this review is unable to provide evidence of causation it is important for healthcare professionals to be aware of these prognostic factors and their incorporation into existing prognostic models [12], which may be useful in clinical practice. However, further research is needed to understand the application of biomarkers in prognostication at the end of life.

Identification of biomarkers of dying is an important area for future research that will lead to both improved clinical tools for managing patients, and shed light on fundamental processes such as the answer to the question: *why do patients die from cancer*?

The “terminal cancer syndrome” theory is characterised by common terminal symptoms including dry mouth, dyspnoea, malnutrition and susceptibility to infection, which are shared amongst heterogeneous groups patients with advanced cancer [7]. In the last three days of life, Bruera *et al*. demonstrated that blood pressure and oxygen saturations decrease significantly in cancer patients [64]. Heart rate variability [72] and autonomic dysfunction [73] have also been described. Thus, changes in vital signs and biomarkers suggest end organ dysfunction.

In non-cancer animal models, McDonald *et al*. demonstrated a rapid loss of body weight and disintegration of the circadian rhythmicity in deep body temperature several days before death in senescent but not presenescent rats [74]. Tankersley *et al*. also demonstrated a predictable sequence of pathophysiological events associated with dying including a fall in daily mean heart rate and a loss of circadian pattern in deep body temperature 3–4 weeks before death [75]. Further, increased lung permeability, reduced lung volume and compliance were seen during a period of terminal senescence [76].

At post-mortem, Kadhim *et al*. demonstrated the over-expression of interleukin-2 (IL-2) *in situ* in brainstem neuronal centres implicated in autonomic control of vital homeostatic functions in adults and children who died from severe illness [77,78]. It was hypothesised that biological stressors trigger over-expression of IL-2 in brainstem neuronal centres, inducing a neurochemical cascade that results in disturbed homeostatic control of cardiorespiratory responses, and eventual death; however, populations were confined to non-cancer diagnoses [78]. Further, Perry *et al*. hypothesised that a neurochemicals including glutamate decarboxylase, tissue pH and tryptophan could reflect novel biomarkers of agonal status [79] and hence the dying phase.

Prognostic factors and post-mortem therefore studies suggest a common biological process to dying. Measurable parameters in the blood suggest a systemic inflammatory response including: elevated CRP, IL-6 and other pro-inflammatory cytokines, hypoalbuminaemia, leucocytosis and neutrophilia. Interestingly, post-mortem studies demonstrated evidence of silent pneumonia in advanced cancer [80,81]. Although it is not possible to assume causation, these findings are of clinical and research interest. For example, the presence of silent pneumonia, may to some extent explain why 24% of patients with terminal cancer have breathlessness despite a lack of risk factors [7]. Further, Kontoyiannis *et al*. demonstrated pulmonary candidiasis in 21% of patients with evidence of pneumonia at post-mortem and 42% of these patients had disseminated candidiasis [82]. *What is the role of immunodeficiency towards the end of life*? *Is there an overarching aetiology for common terminal symptoms*? *Does over-expression of IL-2 in brainstem neuronal centres together with silent pneumonia*, *contribute to breathing changes seen during the dying phase*? *When is the “point of no return” when the administration of antibiotics is futile*? The current lack of research in these areas, together with our desire to improve patient care, highlights the importance for further research into the biology of dying. Biomarkers are important adjuncts in prognostication and the recognition of dying, but equally increase our understanding of the dying process.

Key areas for future research include: serial blood measurements of candidate biomarkers including inflammatory cytokines during the dying phase, intracerebral measurement of cytokines; and correlation with physiological parameters observed during the dying phase. Interestingly, Coyle *et al*. recently presented a feasibility study for taking serial urine samples from hospice inpatients towards the end of life[83] and demonstrated elevated levels of a number of volatile VOCs in the urine during the dying phase[44]. Non-invasive research methodologies are an important area for future research.

### Limitations

There are several limitations with this review. This review only included published studies from year 2000, in order to conduct an in-depth analysis of current studies since the Maltoni *et al*. paper. The authors recognise that a systematic review of randomised controlled trials is the gold standard in research synthesis, however within the constraints of current available evidence, the PRISMA standards for reporting evidence were applied to ensure a systematic structure to the search, selection and review of the literature [13]. However, reviewers were not blinded to the authors, institutions, or journals of publication, which could have introduced selection bias.

This review excluded non-cancer conditions and was limited to patient populations with a median survival of ≤90 days, which meant that a selected number of articles were excluded. This was important given that “*advanced cancer*” is poorly defined in the literature; exclusion of these articles did not change the overall findings of this review.

Many of the studies included in Table 2 were heterogeneous in nature, small and underpowered, and the quality of studies varied considerably. Few studies have specifically investigated changes in the last weeks of life. We screened some studies that may have contained information specific to the objectives of this review, but data was not presented in a way that was specific to advanced cancer. There are considerable ethical implications of conducting research in patients in the last few weeks of life, in particular when this involves invasive procedures. We recognise that this may have limited the number of studies for inclusion in this review. Certainly, a number of included studies were limited by sample size and lack of protocols for collection of blood samples.

It was not possible to provide a synthesis of the evidence due to the heterogeneity of the sample. Despite this, the findings of this review are of particular research interest. This review makes an important contribution to the evidence base on the biology of dying and highlights potential areas for research funding and analysis. Attention has been given to the ethical and methodological implications of research into the biology of dying and future strategies are described.

### Conclusion

The biology of dying is an important area for future research interest. The evidence to date is largely focused on signs, symptoms and prognostic factors. Despite appearances, the extent to which cancer patients follow a common terminal trajectory is uncertain and high quality research should be conducted to explore this concept further. This review identifies a number common themes shared amongst advanced cancer patients and highlights candidate biomarkers which may be indicative of a common biological process to dying. Attention should be placed on understanding the physiological process of dying and identification of candidate biomarkers of imminent death. This will increase clinicians’ confidence in identifying the dying process, inform decision making surrounding end of life care, and ensure the best possible care for patients and their families.

## Supporting information

S1 File PRISMA checklist. (PDF)

S2 File Protocol. (PDF)

## Abbreviations

AGP: α1-acid glycoprotein
ALP: alkaline phosphatase
ALT: alanine aminotransferase
AST: aspartate transaminase
AUC: area under the curve
B12: vitamin B12
BCI: B12 CRP index
BUN: blood urea nitrogen
CACS: cancer anorexia-cachexia syndrome
CAR: CRP/albumin ratio
CPS: Clinicians’ Prediction of Survival
CRP: C-reactive protein
D-PAP: Delirium-Palliative Prognostic Score
ECOG PS: Eastern Cooperative Oncology Group Performance Status
ESR: erythrocyte sedimentation rate
GC-MS: Gas Chromatography Mass Spectrometry
GGT: gamma-glutamyl transpeptidase
GPS: Glasgow Prognostic Score
IFNγ: interferon gamma
IL-10: interleukin-10
IL-12(p70): interleukin-12(p70)
IL-18: interleukin-18
IL-1ra: interleukin-1 receptor antagonist
IL-1β: interleurkin-1 beta
IL-2: interleukin-2
IL-4: interleukin-4
IL-6: interleukin-6
IL-8: interleukin-8
INR: international normalised ratio
JPOS-PI: Japan Palliative Oncology Study-Prognostic Index
KPS: Karnofsky Performance Status
LDH: lactate dehydrogenase
LIF: leukaemia inhibiting factor
MCP-1: monocyte chemoattractant protein-1
m-GPS: modified Glasgow Prognostic Score
MIF: macrophage migration inhibitory factor
MIP-1α: macrophage inflammatory protein-1α
MSSE: mini mental state examination
new-ChPS: new-Chinese Advanced Cancer Patients Scale
NLR: neutrophil-lymphocyte ratio
NMR: Nuclear Magnetic Resonance Spectrometry
NPV: negative predictive value
PaP Score: Palliative Prognostic Score
PINI: Prognostic Inflammatory and Nutritional Index
PiPS: Prognosis in Palliative Care Study
PiPS-A: modified Prognosis in Palliative Care Study-A
PiPS-B: modified Prognosis in Palliative Care Study-B
PPI: Palliative Performance Index
PPV: positive predictive value
PTH: parathyroid hormone
PTHrP: parathyroid hormone related protein
RNA: ribonucleic acid
sTNF- r1: soluble tumour necrosis factor receptor 1
TGF-β1: transforming growth factor β1
TNFα: tumour necrosis factor alpha
TTD: time from initial diagnosis to diagnosis of terminal disease
VOCs: volatile organic compounds
WBC: white blood cell count
WPBAL: WBC/platelet/BUN/AST/LDH prognostic score
WPCBAL: WBC/platelet/CRP/BUN/AST/LDH prognostic score
