# Supplementary material for: A systematically structured review of biomarkers of dying in cancer patients in the last months of life; An exploration of the biology of dying
Source: PLoS One. 2017 Apr 6;12(4):e0175123. doi: 10.1371/journal.pone.0175123 (PMC5383239; doi:10.1371/journal.pone.0175123)
Supplement: S2 File — (PDF) [file pone.0175123.s002.pdf]

Protocol

**Citation (author, year, country)**	
**Purpose of study**	
**Study design according to NICE algorithm**	
**NICE hierarchy of evidence**	Level:
**Hawker appraisal score**	/36:<br><br>1. Abstract and title: /4<br>2. Introduction and aims: /4<br>3. Method and data: /4<br>4. Sampling: /4<br>5. Data analysis: /4<br>6. Ethics and bias: /4<br>7. Findings/results: /4<br>8. Transferability/generalizability: /4<br>9. Implications and usefulness: /4
**Quality assessment for prognostic studies, as described by Maltoni 2005**	/7:<br><br>1. Prospective study: ✓✗<br>2. Well-defined cohort of patients assembled at a common point in the course of their disease: ✓✗<br>3. Random patient selection: ✓✗<br>4. Percentage of patients lost to follow-up 20%: ✓✗<br>5. Ratio between the number of events (death) and the number of potential predictors ≥10: ✓✗<br>6. Prognostic variables fully defined, accurately measured, and available for all or a high proportion of patients: ✓✗<br>7. Reliable measurement of outcome (date of death): ✓✗
**Sample**	
**Sample size**	
**Median survival ≤90 days**	
**Setting**	
**Assessment period (time until death)**	

**Exclusions**	
**Strengths**	
**Weaknesses**	
**Main study findings**	
